# Treatment and analysis of a case of pulmonary embolism with pulmonary infarction following mixed haemorrhoid surgery case report

**DOI:** 10.1097/MD.0000000000050340

**Published:** 2026-08-28

**Authors:** Yanfeng Xu, Yakun Wu, Zhanglong Cong

**Affiliations:** a Department of Anorectal Surgery, Ansteel General Hospital, Anshan, Liaoning, China.

**Keywords:** mixed hemorrhoids, pulmonary embolism, pulmonary infarction

## Abstract

**Rationale:**

Pulmonary embolism following mixed hemorrhoid surgery is uncommon, with few such cases reported in domestic and international literature. This study examines the clinical characteristics, diagnosis, treatment, and outcomes of pulmonary artery embolism after mixed hemorrhoid surgery, aiming to enhance clinicians’ vigilance and prevention of postoperative complications.

**Patient concerns:**

Clinical features of a patient with pulmonary embolism following mixed hemorrhoid surgery, admitted to the Department of Anorectal Surgery at Ansteel General Hospital in June 2025, were analyzed. A 55-year-old female presented with a rectal mass protruding after defecation for approximately 2 weeks. Denies a history of hypertension or diabetes; denies a history of infectious diseases such as hepatitis or tuberculosis; no history of surgery or trauma; no history of varicose veins or swelling and pain in both lower extremities; no history of cardiovascular or cerebrovascular disease or thrombosis; and has had sleep disturbances for over a year. She underwent mixed hemorrhoid surgery on June 24, 2025 and was discharged uneventfully. Five days later, the patient presented to our hospital’s respiratory medicine department with sudden right-sided chest pain accompanied by dry cough. Pulmonary computed tomography (CT) angiography revealed pulmonary embolism with pulmonary infarction. Abnormalities in D-dimer, fibrinogen, and blood gas analysis.

**Diagnoses:**

Combined with the patient’s symptoms and physical findings, led to a definitive diagnosis of pulmonary embolism with pulmonary infarction following mixed hemorrhoid surgery.

**Interventions:**

Following oxygen therapy, anti-inflammatory treatment, and anticoagulation, a follow-up pulmonary CT scan demonstrated reduced pulmonary infarction with improved lesion margins.

**Outcomes:**

Prognosis is favorable, with the patient currently undergoing regular follow-up.

**Lessons:**

Although the risk of pulmonary embolism following hemorrhoid surgery is low, if it does occur and is not promptly diagnosed and treated, the consequences are often severe and may even be life-threatening. This article presents the diagnostic and treatment process for this case and provides analysis and discussion in conjunction with related cases, with the aim of providing some advice for the timely diagnosis and treatment of pulmonary embolism following colorectal surgery.

## 1. Introduction

Pulmonary embolism is the collective term for a group of diseases or clinical syndromes characterized by pulmonary circulatory dysfunction caused by the obstruction of the pulmonary artery and its branching arterioles by endogenous or exogenous emboli.^[1]^ This disease is characterized by a high mortality rate, severe clinical presentation, and acute onset, with an annual incidence of approximately 39 to 115 cases per 100,000 population.^[2]^ It is currently the third leading cause of death from cardiovascular disease worldwide, following only stroke and heart attack.^[3]^ The symptoms and signs of pulmonary embolism lack specificity and may include chest pain, dyspnea, cough, syncope, or hemoptysis. Diagnostic approaches for suspected pulmonary embolism typically involve the Wells score, D-dimer testing, and imaging investigations.^[4]^ Emboli in pulmonary embolism commonly originate from mobile emboli in the right atrium or ventricle, or deep vein thromboses, particularly those arising from the lower extremities.^[5]^ Cases of pulmonary embolism associated with hemorrhoids or following hemorrhoid surgery are relatively rare, leading to a lack of awareness among colorectal surgeons regarding such situations. Due to limited diagnostic and treatment experience, as well as the nonspecific clinical symptoms of pulmonary embolism, misdiagnosis and missed diagnoses are highly likely, which can ultimately lead to serious consequences, or even death.In clinical practice, early and definitive diagnosis of pulmonary embolism remains challenging for internists; for surgeons with relatively limited experience in internal medicine, this presents an even greater challenge. After reviewing the literature, we identified 4 cases of pulmonary embolism associated with hemorrhoids.^[6–9]^ By comparing these cases and incorporating the diagnostic and treatment process of the present case, we hope to raise surgeons’ awareness of the possibility of pulmonary embolism when patients experience respiratory symptoms following hemorrhoid surgery, thereby enabling early diagnosis and treatment.

## 2. Case presentation

### 2.1. Basic information

Patient, female, aged 55, admitted presenting with “an anal mass protruding after defecation for approximately 2 weeks.” On June 24, 2025, underwent combined hemorrhoidectomy and internal ligation under sacral anesthesia, alongside internal hemorrhoid sclerotherapy. The procedure was uneventful. Discharged on July 1, 2025. Five days post-operatively, the patient presented with sudden right-sided chest pain accompanied by a dry cough, exacerbated by deep breathing and coughing, and alleviated in the right lateral decubitus position. She consequently consulted our hospital’s Department of Respiratory Medicine. Medical history: Denied history of hypertension or diabetes mellitus; denied history of infectious diseases including hepatitis or tuberculosis; denied history of blood transfusion; no history of trauma, no history of varicose veins or swelling and pain in both lower limbs, no history of cardiovascular or cerebrovascular disease or thrombosis; has had sleep disturbances for over a year. Physical examination: Temperature: 36.6°C, Pulse: 80 beats per minute, Respiration: 20 breaths per minute, Blood pressure: 110/85 mm Hg. Auscultation revealed clear breath sounds in both lungs with no dry or wet rales heard.

### 2.2. Diagnostic process

**Figure 1.**
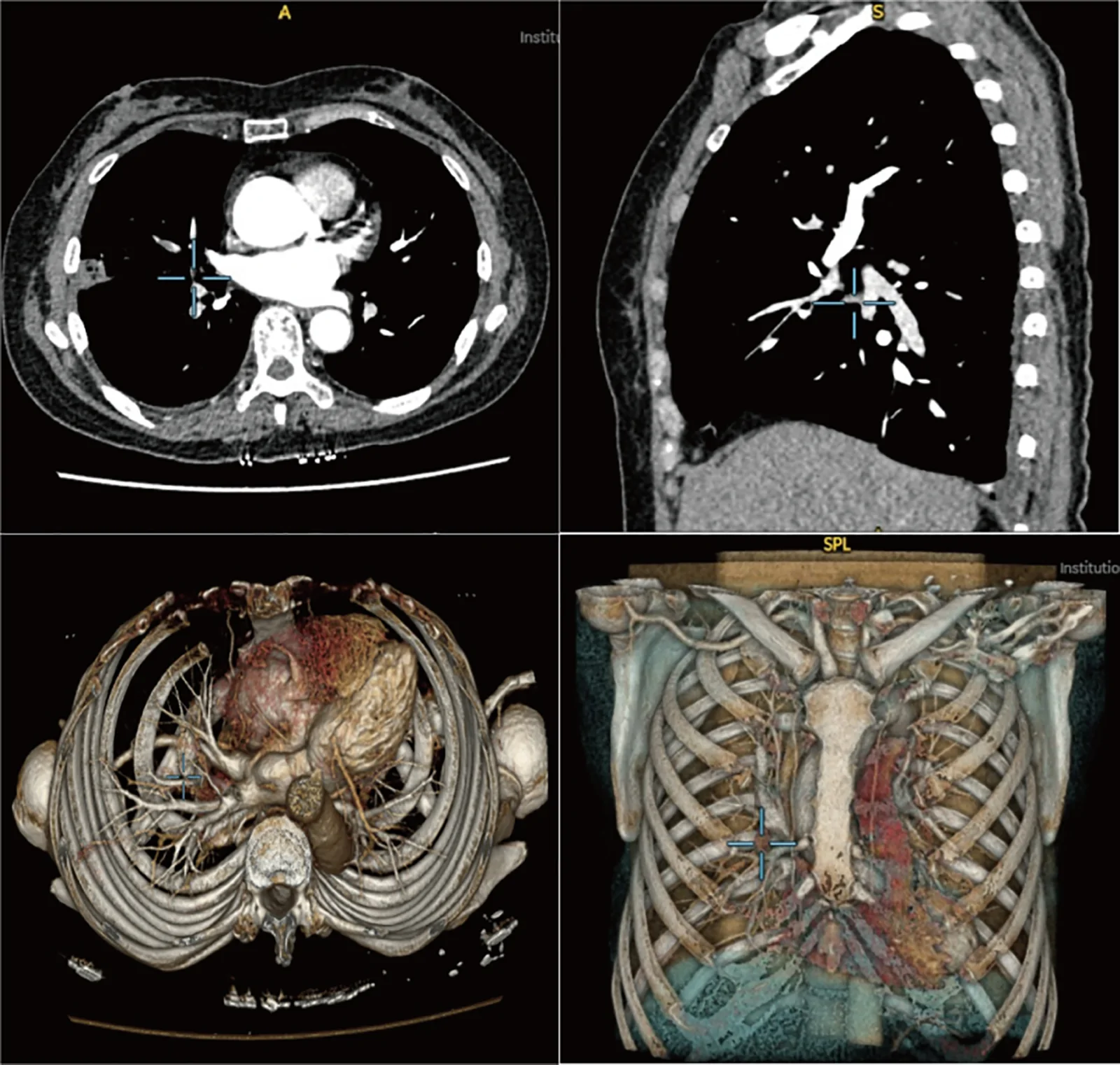
Pulmonary artery CTA results of a patient with mixed hemorrhoids following surgery, dated July 8, 2025. CTA = computed tomography angiography.

Patient admitted on July 6, 2025. Relevant laboratory investigations completed. July 8, 2025: Coagulation panel: Fibrinogen: 5.04 grams/L (↑) D-dimer: 1265.0 ng/mL (↑) July 8, 2025: Arterial blood gas analysis: pH 7.57↑, PaCO_2_ 30.00 mm Hg↓, PaO_2_ 70.00 mm Hg↓, Ca^2+^ 1.12 mmol/L↓, Actual HCO_3_^−^ 27.50 mmol/L↑, Standard HCO_3_^−^ 29.40 mmol/L↑, Hemoglobin concentration 115.00 grams/L↓, Residual alkalinity 5.70 mmol/L↑. Patient exhibits low blood oxygenation with elevated D-dimer levels. The Wells Score, developed by Wells et al in 2000, is based on simple, easy-to-remember clinical symptoms and signs. This scoring system is particularly suitable for the initial screening of patients with pulmonary embolism. The Wells score assesses the likelihood of pulmonary embolism in patients based on the following criteria: history of pulmonary embolism or deep vein thrombosis (1.5 points), heart rate ≥ 100 beats per minute (1.5 points), history of surgery or immobilization within the past 4 weeks (1.5 points), hemoptysis (1.0 point), active malignancy (1.0 point), clinical manifestations of deep vein thrombosis (3.0 points), and other differential diagnoses less likely than pulmonary embolism (3.0 points). The maximum score is 12.5 points. A score < 2 indicates a low probability of pulmonary embolism; 2 ≤ score < 7 indicates a moderate probability; and a score ≥ 7 indicates a high probability of pulmonary embolism.^[10]^ The scores correspond to pulmonary embolism prevalence rates of 3.6%, 20.5%, and 66.7%, respectively, providing important evidence-based support for rapid clinical screening.^[11]^ The patient in this case had a history of surgery within the past 4 weeks, as well as other differential diagnoses with a lower likelihood than pulmonary embolism; the calculated Wells score was 4.5. Pulmonary embolism cannot be excluded. Further investigation with chest computed tomography (CT) and pulmonary artery CT Angiography is indicated. July 8, 2025 Chest CT: Lesion in the middle lobe of the right lung, minor chronic inflammation in the lower lobe of the right lung, small amount of pleural effusion on the right side. Further investigation recommended. July 8, 2025 Pulmonary artery CT angiography: Pulmonary artery embolism in the posterior branch of the middle lobe and the lateral basal branch of the lower lobe of the right lung, with pulmonary infarction in the lateral segment of the middle lobe of the right lung, small amount of pleural effusion on the right side (Fig. 1). July 10, 2025 Electrocardiogram (ECG) findings: Heart rate 56 beats per minute, sinus bradycardia. In conjunction with the patient’s symptoms, physical findings, and medical history, the final diagnosis is pulmonary embolism with pulmonary infarction following mixed hemorrhoid surgery.

### 2.3. Treatment and prognosis

**Figure 2.**
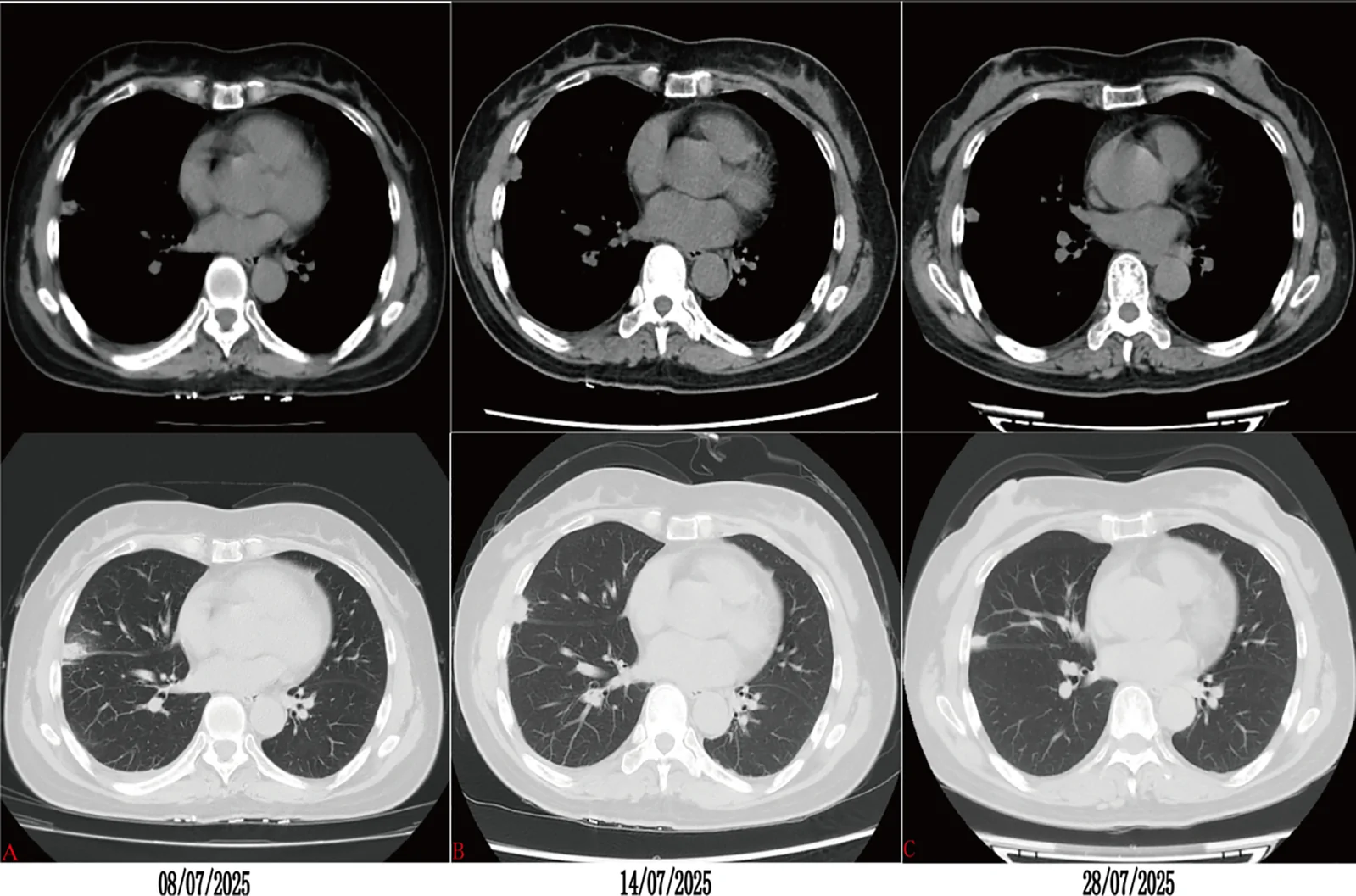
A, B, and C represent the results of 3 pulmonary CT examinations conducted in July 2025 on a patient who underwent mixed hemorrhoid surgery in this case. CT = computed tomography.

Following positive skin tests to cefmetazole sodium for injection and cefoperazone sodium/sulbactam sodium, the patient received intravenous administration of levofloxacin sodium chloride injection and eterimicin sulphate injection for anti-inflammatory treatment over 5 days, alongside oral rivaroxaban tablets for anticoagulation over 5 days. During this period, the patient received supplemental oxygen and was closely monitored for any changes in their condition. On the sixth day of hospitalization, a follow-up chest CT scan revealed: Compared with the chest CT scan dated July 8, 2025, the pulmonary infarction in the middle lobe of the right lung had reduced in size, with clearer lesion margins. Pleural effusion had decreased. The lesion in the lower lobe of the right lung showed partial absorption compared with the previous scan (Fig. 2). On the seventh day of admission, blood tests were repeated. Plasma D-dimer measurement on July 15, 2025: D-dimer was 215.0 ng/mL, which is within the normal range. The patient reported improvement in subjective symptoms.The patient was discharged to continue oral treatment at home with levofloxacin tablets 0.5 grams once daily for one week. A follow-up chest CT scan conducted at the outpatient clinic 2 weeks later revealed: Compared with the plain chest CT scan dated July 14, 2025, pulmonary infarction in the middle lobe of the right lung, with a reduced lesion size compared to the previous scan; localized thickening of the right pleura (Fig. 2). The patient is recovering well.

## 3. Discussion

Pulmonary embolism commonly presents clinically with hypoxemia, hypercapnia, and electrolyte disturbances^[12]^; however, it lacks specificity. Regarding scoring systems: The Wells score is one of the internationally recommended rapid assessment tools for predicting the likelihood of pulmonary embolism. It primarily calculates a score based on the patient’s clinical symptoms, physical signs, and certain basic laboratory test results.^[13]^ It should be noted that the Wells score is not the gold standard for diagnosing pulmonary embolism. In clinical practice, it must be combined with other auxiliary tests and diagnostic methods to achieve an accurate diagnosis of the patient’s condition.^[14]^ In terms of testing: D-dimer is a protein fragment found in the blood. When blood clots dissolve within blood vessels, fibrin breaks down into D-dimers. Consequently, D-dimer levels serve as an indicator of thrombus formation and dissolution within the body. Elevated levels suggest an increased risk of thrombosis^[15]^; however, elevated D-dimer levels are nonspecific, as they may also increase in conditions such as infection, inflammation, trauma, or surgery. Nevertheless, they remain crucial in risk stratification for suspected pulmonary embolism,^[2]^ to help assess the risk of pulmonary embolism and determine whether further diagnostic investigations are required. In terms of examination: most cases present with nonspecific electrocardiographic abnormalities.^[16]^ Pulmonary CT angiography is the preferred imaging modality for high-risk patients or those with clinically suspected pulmonary embolism and elevated D-dimer levels. It offers high sensitivity and specificity for diagnosing pulmonary embolism, being noninvasive, convenient, and offers high specificity and sensitivity. It clearly visualizes thrombi within the pulmonary artery and its branches, pinpoints the location and extent of embolism, assesses right ventricular load and function, and excludes other pulmonary conditions (such as aortic dissection or pneumothorax).^[17]^ The most commonly used diagnostic tests for confirmation and exclusion.^[18]^ Treatment regimen: This encompasses anticoagulant therapy, thrombolytic therapy, interventional and surgical interventions, and respiratory and circulatory support. According to the Wells criteria, treatment for moderate-to-high-risk pulmonary embolism in China currently remains primarily anticoagulant therapy and intravenous thrombolysis.^[19]^ Unless the patient has contraindications to anticoagulation, anticoagulant therapy should be initiated immediately upon suspicion of pulmonary embolism.^[20]^

We searched PubMed, China National Knowledge Infrastructure, Wanfang, and VIP databases using the keywords “hemorrhoids,” “pulmonary embolism,” and “case report” to identify case reports published through July 2026 regarding pulmonary embolism occurring in patients with hemorrhoids or following hemorrhoid surgery. We also screened the references in the identified papers to find additional cases for comparison of clinical manifestations, treatment, and outcomes. Following a thorough review and screening process, we included 4 articles reporting a total of 4 cases.^[6–9]^ The case data are presented in Table 1.

**Table 1:** A summary of existing case reports on pulmonary embolism in patients with hemorrhoids and following hemorrhoid surgery.

References	Age	Sex	Clinical manifestations	Basis for diagnosis	Treatment	Results
Acute pulmonary embolism^[6]^	42	M	On the 4th day after injection treatment for internal hemorrhoids, the patient experienced chest pain, shortness of breath, and a dry cough; both lower limbs were swollen and distended, with tenderness in the gastrocnemius muscles	Pulmonary artery CTA: Multiple filling defects in both pulmonary arteries; Doppler ultrasound of both lower extremities showed no thrombi; ECG: sinus rhythm, possible left ventricular hypertrophy; Elevated D-dimer levels	Low-molecular-weight heparin calcium plus rivaroxaban for anticoagulation; anti-infective therapy	Survived
Acute multiple pulmonary embolism^[7]^	47	F	Half an hour after laser hemorrhoidectomy, the patient experienced chest pain, wheezing, and vomiting; blood pressure was 75/60 mm Hg; cyanosis of the lips and fingers was observed	X-ray showed multiple patchy shadows in both lungs; arterial blood gas analysis: partial pressure of oxygen 40.2 mm Hg; ECG: reduced amplitude of wall motion in the left ventricular anterior wall and interventricular septum; electrocardiogram: transient ST-segment depression; positive D-dimer	Urokinase thrombolysis + low-molecular-weight heparin anticoagulation; dopamine + methoxamine for blood pressure elevation; Antibiotic therapy	Survived
Bilateral pulmonary embolism^[8]^	72	M	Following a recurrence of hemorrhoids (without surgery): shortness of breath, chest pain, and hemoptysis; fever of 38.3°C; respiratory rate of 30 breaths per minute; cyanosis of the lips	Pulmonary ventilation-perfusion scan: bilateral pulmonary embolism; echocardiogram: dilation of the right lower pulmonary artery; ECG: sinus tachycardia, ST-T changes; blood gas analysis: partial pressure of oxygen 46 mm Hg; 3 sputum samples tested negative for mycobacterium tuberculosis and tumor cells	Urokinase + low-molecular-weight heparin + warfarin anticoagulation	Survived
Acute pulmonary embolism^[9]^	78	M	On the 4th day after undergoing a combined external excision and internal ligation procedure for mixed hemorrhoids plus an injection procedure for internal hemorrhoids, the patient fainted, became unconscious, experienced respiratory arrest, cardiac arrest, undetectable blood pressure, and generalized cyanosis	ECG: inferior wall myocardial infarction; elevated cardiac enzymes	Endotracheal intubation, cardiopulmonary resuscitation; transferred to a higher-level hospital, where follow-up confirmed acute pulmonary embolism, and thrombolytic therapy was administered	Survived

CTA = computed tomography angiography.

Including the cases of pulmonary embolism associated with the exacerbation of hemorrhoids and those occurring after hemorrhoid surgery, as well as the cases in this study, there were a total of 5 patients – 2 women and 3 men – ranging in age from 42 to 78 years, with a median age of 55 years. The similarities among the cases are as follows: None had a history of lower extremity varicose veins or swelling and pain in both lower extremities, nor did they have a history of cardiovascular or cerebrovascular disease or thrombosis. All patients experienced symptoms of chest discomfort following an exacerbation of hemorrhoids – both before surgery and after surgery – and were diagnosed with pulmonary embolism through relevant tests and examinations. Ultimately, all patients recovered and were discharged after receiving symptomatic treatment, including thrombolysis. Differences among the cases are as follows: Some patients may present with clinical symptoms such as swelling and distension of both lower limbs, tenderness of the gastrocnemius muscles, decreased blood pressure, fever, reduced partial pressure of oxygen on arterial blood gas analysis, and increased respiratory rate.

An analysis of the clinical similarities and differences among such patients suggests that when patients experience the symptoms described in the above case following hemorrhoid surgery, clinicians should be on high alert and promptly conduct relevant tests and examinations to diagnose pulmonary embolism at an early stage, thereby ensuring that patients receive appropriate treatment as soon as possible.

Relevant studies indicate that if chest discomfort occurs within one week after surgery, one should be highly vigilant for the possibility of a pulmonary embolism.^[21]^ The median time to occurrence of pulmonary embolism following surgery was 9 days.^[22]^ This case of pulmonary embolism occurred following mixed hemorrhoid surgery, which is relatively uncommon. Hemorrhoidal masses frequently induce local intravascular thrombosis due to damage to the overlying mucosa or skin and chronic inflammatory responses. After surgery, if a dislodged blood clot enters the inferior vena cava via an anastomotic branch between the rectal venous plexus and the inferior vena cava, and is subsequently carried by the blood flow to the pulmonary artery, it may cause a pulmonary embolism and result in a pulmonary infarction. Taking all factors into account, it is highly likely that the embolus in this case originated from a thrombus that had dislodged from a hemorrhoidal vein. However, the patient’s insufficient physical activity following surgery, combined with factors such as poor sleep quality and anxiety, failed to effectively prevent thrombus formation. Furthermore, since the possibility of tuberculosis and cancer has not yet been ruled out, the source of the emboli causing the pulmonary embolism may not be singular. These circumstances should also be considered as potential factors contributing to the pulmonary embolism. The patient was assessed as intermediate risk according to the Wells score. Following oxygen therapy, anti-inflammatory treatment, and oral anticoagulation, D-dimer levels normalized prior to discharge. At the two-week follow-up, the repeat chest CT scan demonstrated marked improvement compared to the initial scan, indicating favorable recovery.^[23]^

This case lacks preoperative laboratory data on the patient’s D-dimer levels and blood gas analysis. Furthermore, screening results for conditions such as tuberculosis, cancer, and lower extremity thrombosis are missing. Therefore, there are certain limitations in determining the source of the embolus in this case of pulmonary embolism. However, given that 4 previous cases of pulmonary embolism following hemorrhoid flare-ups or hemorrhoid surgery have been reported, and after reviewing the diagnostic and treatment processes of these cases, we believe that the diagnostic methods and treatment strategies for postoperative pulmonary embolism following hemorrhoid surgery described in this article are worthy of surgeons’ attention and consideration in clinical practice.

## 4. Conclusion

In summary, pulmonary embolism following mixed hemorrhoid surgery is relatively uncommon, with few reports both domestically and internationally. If not promptly diagnosed and treated, pulmonary embolism often leads to severe consequences. Identifying rare etiologies and improving the diagnostic rate of pulmonary embolism in clinical practice are particularly crucial. We hope that clinicians will remain vigilant and give this matter the attention it deserves.

## Author contributions

**Conceptualization:** Yanfeng Xu.

**Data curation:** Zhanglong Cong.

**Formal analysis:** Zhanglong Cong.

**Investigation:** Yanfeng Xu.

**Methodology:** Zhanglong Cong.

**Project administration:** Zhanglong Cong.

**Supervision:** Yakun Wu.

**Validation:** Zhanglong Cong.

**Visualization:** Yanfeng Xu.

**Writing – original draft:** Yanfeng Xu.

**Writing – review & editing:** Zhanglong Cong.
